# Mining and evaluation of security alert signals of neuroprotective agents based on the Jinan adverse event reporting system database: a retrospective study

**DOI:** 10.3389/fphar.2025.1529923

**Published:** 2025-08-05

**Authors:** Yu-yao Guan, Jing Yang, Ying-mei Qi, Chao Song, Lei Zheng

**Affiliations:** ^1^ Department of Pharmacy, Shandong Provincial Third Hospital, Cheeloo College of Medicine, Shandong University, Jinan, Shandong, China; ^2^ Jinan Adverse Drug Reactions and Medical Device Adverse Event Monitoring Center, Jinan, Shandong, China

**Keywords:** security alert signals, neuroprotective agents, data mining, adverse event, stroke

## Abstract

**Background:**

Pharmacologic agents with proposed neuroprotective properties are increasingly investigated; however, limited regulation and heterogeneous evidence raise concerns about their safety profiles. To establish a foundation for its safe clinical use, the data mining technology was used to investigate the safety warning signals of neuroprotective agent post-market approval.

**Methods:**

The Jinan adverse event (AE) reporting system database was searched for AEs related to neuroprotective agents from January 2000 to March 2022. Basic information pertaining to patients, reports, and common AEs was analyzed. Subgroup analyses were performed to examine the distribution of adverse reaction signals for each drug across different age groups and genders. Sensitivity analyses were conducted by stratifying patients based on concomitant medication use to evaluate the distribution of adverse reaction for each drug under different stratification conditions. Kaplan–Meier curves of time were used to analyze AEs for each drug. Four disproportionality analysis methods, namely, proportional reporting ratio (PRR), reporting odds ratio (ROR), Bayesian Confidence Propagation Neural Network (BCPNN), and the Medicines and Healthcare products Regulatory Agency (MHRA), were applied to obtain alert signals for this class of drugs. We further examined the presence of the detected signals on medicine labels in China and two developed countries (USA and Japan).

**Results:**

In total, 168,314 AEs were reported, of which 2,094 were associated with neuroprotective agents. Risk signals demonstrated significant variations across age groups, gender strata, and analyses with/without concomitant medication adjustments. For the following medications—citicoline sodium, troxerutin and cerebroprotein hydrolysate, vinpocetine, *Ginkgo biloba* leaf extract, and cerebroprotein hydrolysate injection—50% of AEs occurred before the median time point. Using the PRR, ROR, MHRA, and BCPNN methods, 81, 57, and 68 signals were detected, respectively. Among the 81 signals, 30 AEs were not included on the drug labels used in China. Of these, 11 AEs were not included on the drug labels used in Japan; two AEs were included on the drug labels used in China but not in Japan.

**Conclusion:**

The Jinan AE reporting system database used to mine warning signals can be used to analyze AEs after the marketing of neuroprotective agents, thereby reducing the risk associated with their clinical use.

## 1 Introduction

Stroke constitutes a major public health threat in China, with epidemiological data ranking its incidence and mortality among the highest globally. Nationwide statistics (2021–2022) reveal an age-standardized stroke incidence of 338.6 per 100,000 among adults ≥18 years, with notable urban–rural mortality disparities (urban: 140.02/100,000 *vs*. rural: 175.58/100,000) (Chinese Cardiovascular Health and Disease, 2023). A concerning trend of young-onset condition has been documented. This disease burden has driven widespread use of neuroprotective agents—pharmacologic interventions targeting the modulation of ion channels, free radical scavenging, and metabolic activation to mitigate neurological deficits (Paul and Candelario-Jalil, 2021; Dammavalam et al., 2024; Haupt et al., 2023). Nevertheless, the clinical value of neuroprotective agents remains controversial. International guidelines such as the American Heart Association/American Stroke Association (AHA/ASA) consistently emphasize the lack of high-quality evidence supporting the efficacy of these agents. China stands as the sole nation that incorporates these agents with a Class I Level B recommendation into its guidelines for acute ischemic stroke management. This divergence has created a distinctive practice pattern that contrasts markedly with prevailing global therapeutic norms. Neuroprotective agents represent a broad therapeutic category referenced in the Chinese Guidelines for Diagnosis and Treatment of Acute Ischemic Stroke (2023). Among these, edaravone, citicoline, and *Ginkgo biloba* extracts are explicitly recommended in China’s primary stroke prevention guidelines. Other agents—including oxiracetam, cerebroprotein hydrolysate, troxerutin and cerebroprotein, calf serum deproteinized extract, vinpocetine, and cattle encephalon glycoside and ignotin—are included in secondary stroke prevention guidelines. Notably, monosialotetrahexosylganglioside (GM1) and mouse-derived nerve growth factor (mNGF), although not endorsed in either primary or secondary stroke prevention guidelines, are frequently prescribed off-label for stroke patients in clinical practice.

In conjunction with the widespread use of neuroprotective agents in China, serious adverse reactions have occurred frequently, with drug safety concerns becoming increasingly prominent (Zhu et al., 2022; Wang et al., 2025; Cao and Shi, 2019). Consequently, it is imperative to investigate the risk signals of neuroprotective agents after marketing. The Chinese healthcare system implemented the First National List of Key Drugs for Rational Use Monitoring (2019) in response to growing concerns about inappropriate use of medications in clinical practice. This policy initiative specifically targeted medications with (1) high prescription volumes; (2) significant expenditure impacts; (3) potential safety concerns. Neuroprotective agents were prominently featured in this catalog due to their widespread off-label use in neurological disorders and limited high-quality evidence supporting therapeutic benefits, creating an urgent need for systematic pharmaco-vigilance investigations. Eleven neuroprotective agents were selected from China’s First National List of Key Drugs for Rational Use Monitoring to investigate their adverse drug reaction risks. The 11 neuroprotective agents evaluated in the present study exert their effects through diverse mechanisms, including membrane stabilization, metabolic activation, free radical scavenging, and multi-target synergistic actions, yet demonstrate significant variability in clinical applications. Agents such as edaravone, *Ginkgo biloba* leaf extract, vinpocetine, and deproteinized calf blood serum extract primarily target acute-phase neuroprotection through ROS scavenging and microcirculation improvement. In contrast, monosialotetrahexosylganglioside sodium, mouse nerve growth factor, cerebroprotein hydrolysate, troxerutin–cerebroprotein complex, and cattle encephalon glycoside and ignotin facilitate structural repair through neurotrophic factor-mediated axonal regeneration. For chronic-phase management, oxiracetam and citicoline enhance cognitive function by modulating cholinergic transmission and phospholipid metabolism. This highlights the spectrum of therapeutic mechanisms among these neuroprotective agents.

Neuroprotective agents are less recognized worldwide, according to certain reports; among the 11 national guidelines in Europe, America, and Asia, only China uses neuroprotectants as clinically recommended drugs, four countries do not recommend them, one country has insufficient evidence and only uses them for clinical trials, and five countries do not mention the application of neuroprotective agents (Wang et al., 2016). In China, the Chinese Guidelines for the Diagnosis and Treatment of Acute Ischemic Stroke 2023 recommends use of neuroprotective agents: the efficacy and safety of neuroprotective agents warrant further confirmation through more high-quality clinical trials (Class I recommendation, Level B evidence) (Chinese Society of Neurology and Cerebrovascular Group, 2024). Therefore, it is necessary to evaluate its post-marketing safety to provide some basis for the rationality of the application of neuroprotective agents.

Neuroprotective agents such as monosialotetrahexosylganglioside sodium, mouse nerve growth factor, edaravone, cattle encephalon glycoside and ignotin, troxerutin and cerebroprotein hydrolysate, cerebroprotein hydrolysate, oxiracetam, vinpocetine, deproteinized calf blood serum, citicoline sodium, and *Ginkgo biloba* leaf extract are used in large amounts in our country. Adverse event (AE) reporting systems are an important source of data for research on drug safety, and post-marketing safety on these data using data mining techniques is gradually being studied. The Jinan AE reporting system monitoring system was established in 2000, collecting AE spontaneously reported by medical institutions and pharmaceutical companies in Jinan.

The risk signal mining technology in pharmaco-vigilance is of great significance for ensuring drug safety (Kumar, 2024; Wang et al., 2024; Scosyrev et al., 2025). In this study, we used data from the Jinan AE reporting system to detect the signals of 11 neuroprotective agents using the risk-signal mining technology. The signals were compared with drug labeling information from China, the United States, and Japan. This study aims to provide a reference for the safe clinical use of 11 neuroprotective agents.

## 2 Materials and methods

### 2.1 Selection criteria for neuroprotective agents

The agents were selected from the First National List of Key Drugs for Rational Use Monitoring issued by China’s National Health Commission and are clinically applied for treating neurological damage in stroke.

### 2.2 Database and study drug

The adverse drug reaction (ADR) data in the Jinan ADR Reporting System are electronically collected from healthcare institutions and pharmaceutical manufacturers. Database access is restricted; approval from the Jinan ADR Monitoring Center is required for data use. The database established by the Jinan AE reporting system was selected for this study. The reports pertaining to 11 neuroprotective agents from January 2000 to March 2022 were searched. The database had a total of 168,314 reports, of which 2,094 reports were related to neuroprotective agents. The reports included information such as patient age, gender, suspected drug information, concomitant drug use, time of occurrence of adverse reaction, and the name of the adverse reaction.

Drug names were coded using the Anatomical Therapeutic Chemical Classification system (ATC codes), and AEs were coded using preferred terms (PTs) from the World Health Organization Adverse Reaction Terminology (WHO-ART). We generated drug–AE pairs for each report using the ATC code of the reported drug and the WHO-ART PT of the AEs, on which all descriptive statistics and data mining were based. Since multiple AEs were reported for the same patient, there were 3,250 drug–AE pairs associated with neuroprotective-targeted therapeutic agents.

The criteria for defining serious adverse drug reactions (SADRs) are established in accordance with the Adverse Drug Reaction Reporting and Monitoring Management Regulations issued by China’s National Health Commission, which fully adopts the ICH E2A guidelines.

### 2.3 Statistical analysis

Disproportionality measurement, also known as a proportional imbalance measurement, is a data mining technique currently used to identify adverse drug reactions. The method is based on a four-compartment table of ADR events (Table 1). If the number of occurrences of an association between a drug and an AE is greater than the expected number of occurrences, it is indicative of a proportional imbalance, suggesting that the probability of the drug and the AE co-occurring within a single report surpasses that of the other drug and the other AE co-occurring in a single report. Consequently, it can be assumed that there are some potential associations between the drug and the AE and that an ADR signal may be detected.

**TABLE 1:** Two-by-two contingency table.

	Target ADR	Other ADR	Total
Target drugs	A	B	A+B
Other drugs	C	D	C+D
Total	A+C	B+D	A+B+C+D

Proportional imbalance measurements are classified into frequency and Bayesian methods. Common frequency methods include the proportional reporting ratio (PRR) (Sakaeda et al., 2013), the reporting odds ratio (ROR) (Peng et al., 2020), and the combined criterion method [Medicines and Healthcare products Regulatory Agency (MHRA) or Multicriteria Analysis (MCA)] (Scosyrev et al., 2025). Bayesian methods include the Bayesian Confidence Propagation Neural Network (BCPNN) method (Fukazawa et al., 2018). According to the corresponding calculation formula and signal generation conditions, the signals of drugs and AEs were detected (Table 2).

**TABLE 2:** Signal data mining algorithms and their signal-generating satisfied conditions.

Index	Signal-generating satisfied condition	Definition
PRR	PRR 95% CI > 1, and *n* ≥ 3	*PRR = [A/(A+B)]/[C/(C+D)*, *SE*(ln*PRR*) *= [(1/A-1/(A+B)+1/C-1/(C+D)]* ^*−1/2*^, 95% CI = *e* ^ln*(PRR)±1.96SE(*ln*PRR)*^
ROR	PRR 95% CI > 1, and *n* ≥ 3	*ROR=(A/C)/(B/D)=(AD)/(BC)*, *SE*(ln*ROR*) = *(1/A+1/B+1/C+1/D)* ^*−1/2*^, 95% CI = e^ln*(ROR)*±*1.96SE*(ln*ROR*)^
MHRA	PRR ≥2, A ≥ 3, χ^2^ ≥ 4	*PRR = [A/(A+B)]/[C/(C+D)], χ* ^*2*^ *=[(|AD-BC|-n/2)^2^n]/[(A+B)(A+C)(C+D)(B+D)]*
BCPNN	IC 95% CI lower limit >0	*IC=log*_2_ *[p(x,y)]/[p(x)p(y)]=log*_2_ *[A(A+B+C+D)]/[(A+B)(A+C)], IC=E(IC_ij_), SD=[V(IC_ij_)]* ^*−1/2*^, *γ*_ij_ *= 1, α*_i_ *= β*_j_ *= 1, α = β = 2, c*_ij_ *= A, c*_*i*_ *= A+B, c*_*j*_ *= A+C, N = A+B+C+D* *γ = γ*_*ij*_ *[(N+ α)(N+β)]/[(c_i_+ α*_*i*_ *)(c*_*j*_ *+β*_*j*_ *)],* *E(IC*_*ij*_ *)=log2[(c*_*ij*_ *+γ*_*ij*_ *)(N+α)(N+β)]/[(N+γ)(c*_*i*_ *+α*_*i*_ *)(c*_*j*_ *+β*_*j*_ *)] = lo* *g* *2[(c*_*ij*_ *+* *γ_ij_)γ]/(N+γ),* *V(IC*_*ij*_ *)={[(N-c*_*ij*_ *+γ-γ*_*ij*_ *)/((c*_*ij*_ *+γ*_*ij*_ *)(1+N+γ)]+[(N-c*_*i*_ *+α-α*_*i*_ *)/((c*_*i*_ *+α*_*i*_ *)(1+N+α)]+[(N-c*_*j*_ *+β-β*_*j*_ *)/(c*_*i*_ *+β*_*j*_ *)(1+N+β)]}/(log2)* ^*2*^

Note: IC, information score; SD, standard deviation; CI, confidence interval.

AEs can be defined as a signal detected through any of the four indicators: PRR, ROR, BCPNN, or MHRA. The signals detected in the Jinan AE reporting system were compared with information from drug inserts in China and in both the United States and Japan. All statistical analyses were performed using SPSS 20.0 and Microsoft Excel^®^ 2010.

The BCPNN method was used as a reference standard for determining false positives, false negatives, true positives, and true negatives and comparison of their sensitivity, specificity, positive predictive value, negative predictive value, and the Jordan index (Table 3).

**TABLE 3:** Four-fold table for calculating the sensitivity and specificity.

	BCPNN (+)	BCPNN (−)	Total
Other method (+)	a	b	a+b
Other method (−)	c	d	c+d
Total	a+c	b+d	a+b+c+d

Note: sensitivity = a/(a+c); specificity = d/(b+d); positive predictive value = a/(a+b); negative predictive value = d/(c+d); Jorden index = [a/(a+c)]+[d/(b+d)]-1.

Subgroup analyses were performed to examine the distribution of adverse reaction signals for each drug across different age groups and genders. The median time point used in time-to-onset analyses is measured from drug initiation to AE onset. It is calculated specific to each agent. To evaluate the distribution of adverse reaction signals for each drug under different stratification conditions, sensitivity analyses were conducted by stratifying patient data based on concomitant use of medication.

## 3 Results

**FIGURE 1:**
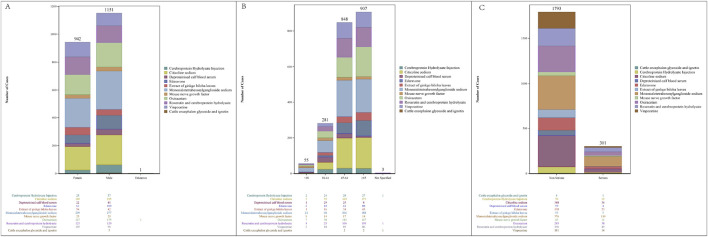
Characteristics of reports associated with neuroprotective agents from 2000 to 2022. **(A)** Sex distribution of AE reports; **(B)** age distribution of AE reports; **(C)** serious report distribution of AE reports.

Neuroprotective agent reports were categorized based on their characteristics (Figure 1). Of the 2,094 reports, 1,151 (54.97%) were from male patients and 942 (44.99%) from female patients. According to age, patients aged >65 years (43.31%) had the highest frequency of AEs, followed by those aged 45–64 years (40.50%), 18–44 years (13.42%), and lastly, <18 (2.63%) years, who had the lowest frequency. Non-serious reports accounted for approximately 75% of cases, reflecting a 3:1 ratio compared to serious reports.

**FIGURE 2:**
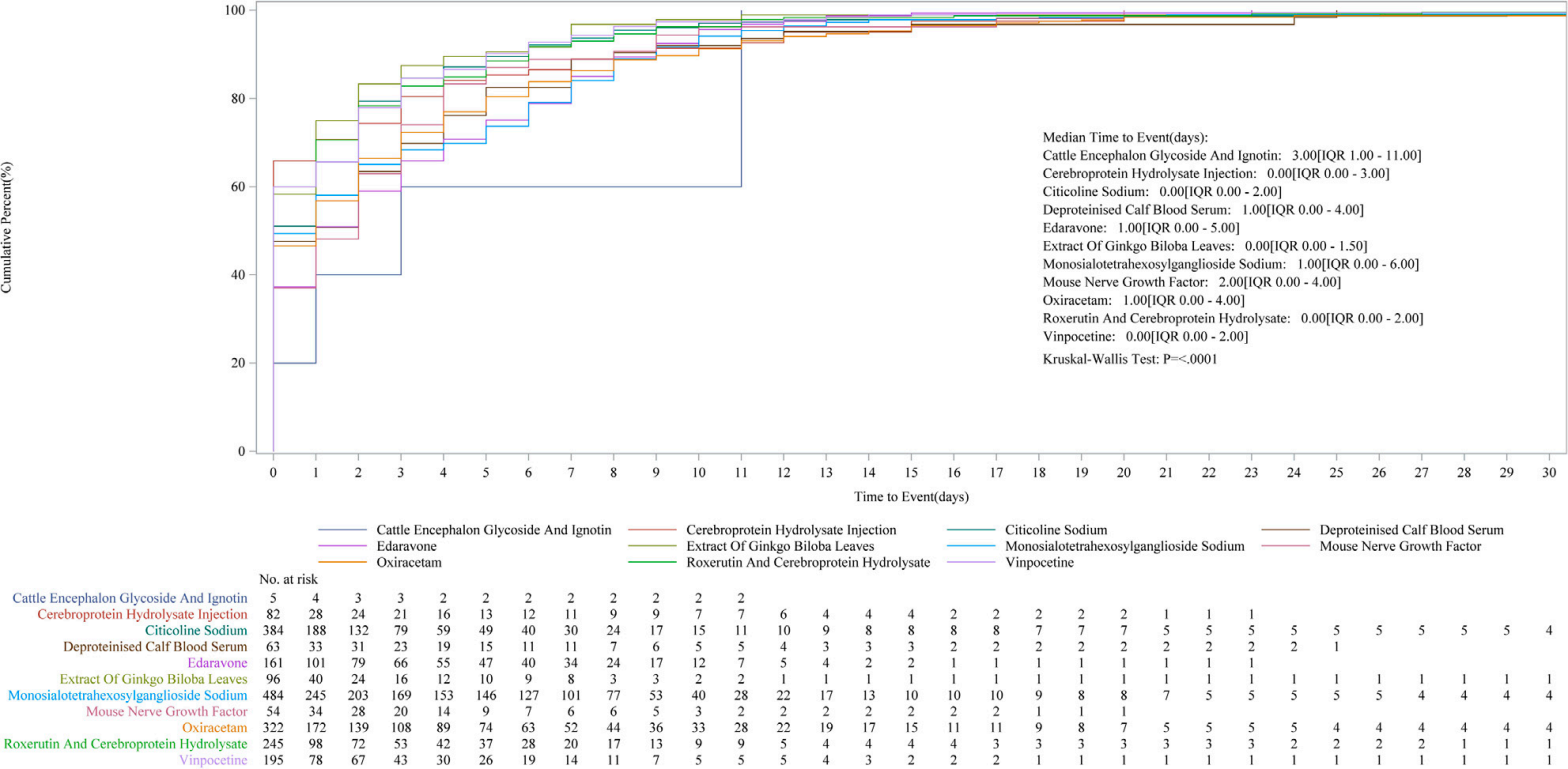
Kaplan–Meier curves of time to AEs for each drug (showing cumulative incidence over time).

Cumulative incidence curves comparing the timing of AE post-medication showed no statistically significant differences (Figure 2).

For the following medications—citicoline sodium, troxerutin and cerebroprotein hydrolysate, vinpocetine, *Ginkgo biloba* leaf extract, and cerebroprotein hydrolysate injection—50% of AEs occurred before the median time point, suggesting that enhanced safety monitoring should be prioritized before this critical time.

**FIGURE 3:**
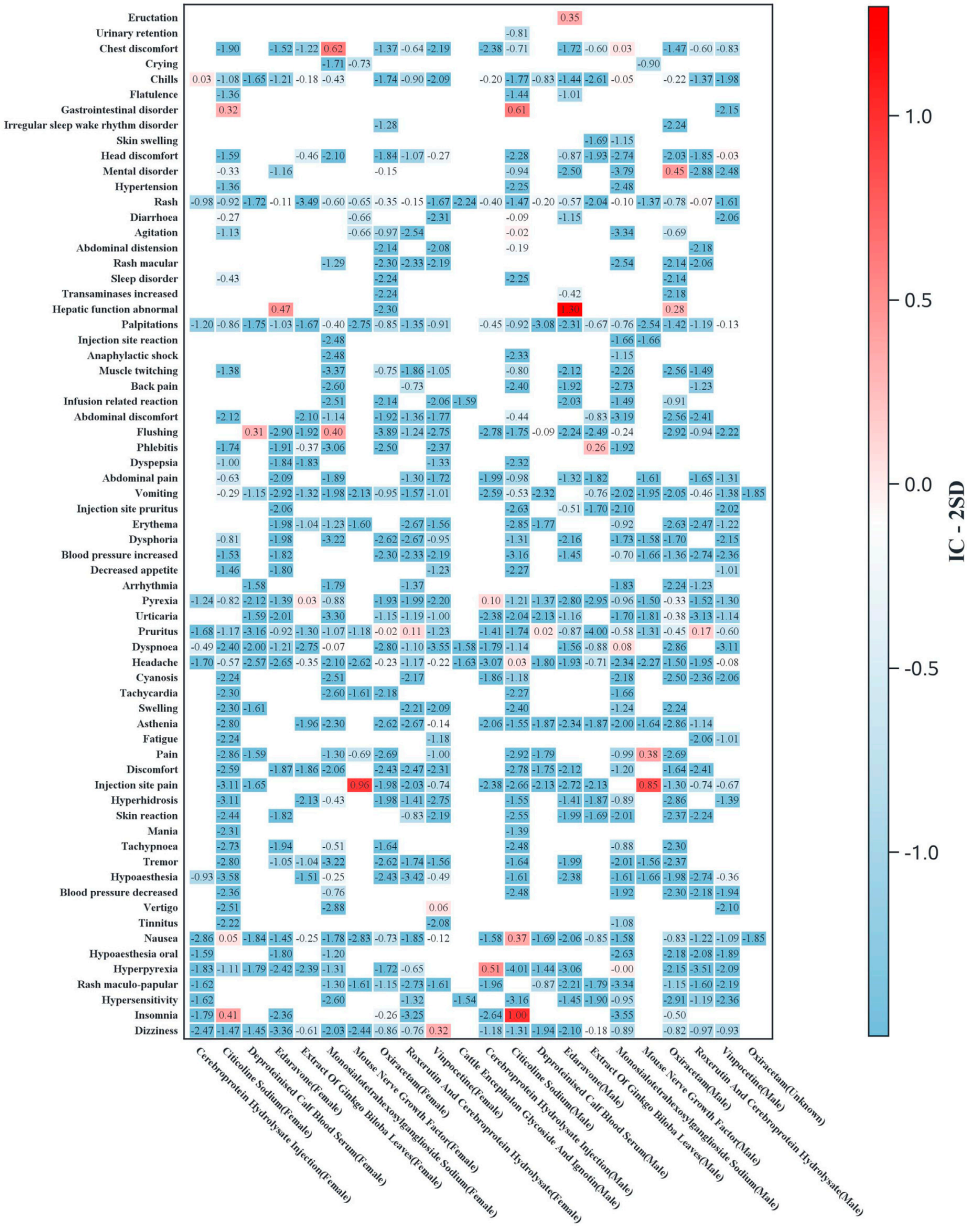
Heatmap of the adverse reaction signal distribution by drug and gender (the *Y*-axis displays preferred terms for AEs, limited to terms with ≥3 cases for any drug; the *X*-axis shows drug names stratified by gender. Heatmap values represent IC-2SD, with darker colors indicating stronger signals).

**FIGURE 4:**
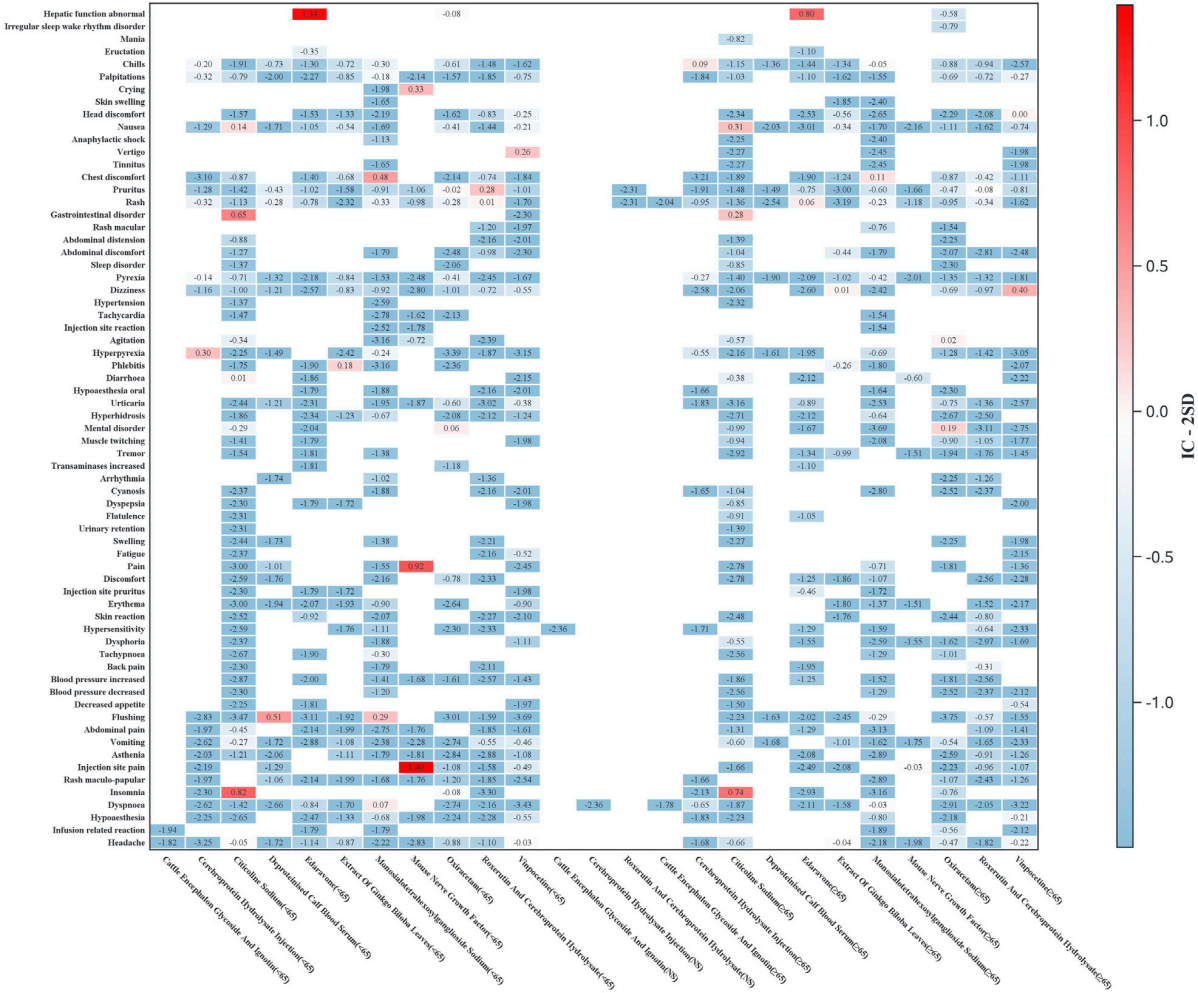
Age-stratified heatmap of ADR signals (*Y*-axis: preferred terms with ≥3 cases for any drug; *X*-axis: drug names by age subgroups. Heatmap values represent IC-2SD, with darker hues indicating stronger signal strength).

**FIGURE 5:**
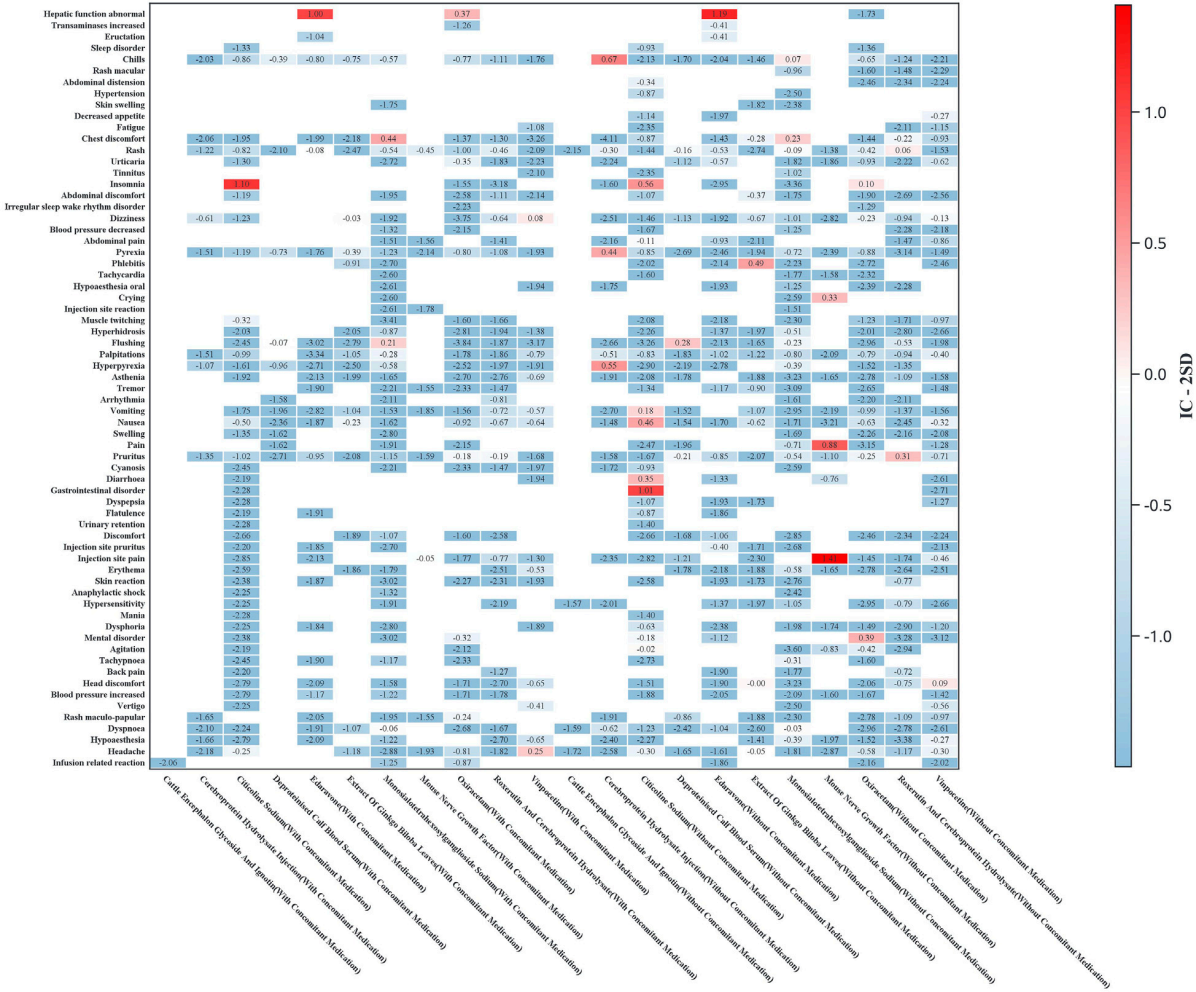
Heatmap of ADR signals stratified by concomitant medication use (*Y*-axis: preferred terms with ≥3 reported cases for any drug; *X*-axis: drug names stratified by concomitant medication status. The color intensity represents IC-2SD values, with darker shades indicating stronger signals).

Subgroup analyses were conducted to evaluate ADR signal distributions stratified by sex (Figure 3) and age (Figure 4). Sensitivity analyses were performed by stratifying patients based on concomitant medication use to examine drug safety signal patterns across these strata (Figure 5).

Gender-stratified analysis revealed significant male predominance in neuropsychiatric (e.g., citicoline-associated insomnia) and systemic reactions (e.g., hyperpyrexia with cerebroprotein hydrolysate). Notably, flushing signals (monosialotetrahexosylganglioside and deproteinized calf blood extract) and local reactions (mouse nerve growth factor) were consistently stronger in male patients.

Age-stratified analysis revealed distinct safety profiles. Younger patients (<65 years) showed significant signals for neuropsychiatric (e.g., citicoline-associated insomnia) and systemic reactions (e.g., hyperpyrexia with cerebroprotein hydrolysate), while the elderly (≥65 years) exhibited higher risks of nausea (citicoline) and dizziness (vinpocetine).

The top five most frequently reported adverse reactions associated with the 11 neuroprotective agents (see Table 4) may serve as clinical safety warnings for medication use.

**TABLE 4:** The top five most frequently reported adverse reactions associated with the 11 neuroprotective agents.

Rank	Oxiracetam	Citicoline sodium	Monosialotetrahexosylganglioside sodium	Cerebroprotein hydrolysate	Troxerutin–cerebroprotein complex	Deproteinized calf blood serum	Edaravone	Extract of *Ginkgo biloba* leaves	Vinpocetine	Mouse nerve growth factor	Cattle encephalon glycoside
1	Pruritus	Nausea	Rash	Rash	Rash	Rash	Rash	Nausea	Nausea	Injection site pain	Headache
2	Rash	Rash	Chest discomfort	Chills	Pruritus	Pruritus	Liver function abnormal	Headache	Pruritus	Rash	Rash
3	Chills	Insomnia	Pruritus	Pyrexia	Chest discomfort	Chills	Pruritus	Chest discomfort	Rash	Pruritus	Dyspnoea
4	Nausea	Dizziness	Chills	Pruritus	Chills	Flushing	Chills	Chills	Headache	Crying	Anaphylactoid reaction
5	Headache	Vomiting	Flushing	Palpitations	Nausea	Nausea	Chest discomfort	Pyrexia	Dizziness	Pain	Infusion-related reaction

Deproteinized calf blood serum	Edaravone	Extract of *Ginkgo biloba* leaves	Vinpocetine	Mouse nerve growth factor	Cattle encephalon glycoside
Rash	Rash	Nausea	Nausea	Injection site pain	Headache
Pruritus	Liver function abnormal	Headache	Pruritus	Rash	Rash
Chills	Pruritus	Chest discomfort	Rash	Pruritus	Dyspnoea
Flushing	Chills	Chills	Headache	Crying	Anaphylactoid reaction
Nausea	Chest discomfort	Pyrexia	Dizziness	Pain	Infusion-related reaction

In the present study, we detected 81 signals using the PRR method, 81 signals using the ROR method, 57 signals using the MHRA method, and 68 weak signals using the BCPNN method (Supplementary Table S1). The BCPNN method produced 51 weak signals, 17 medium signals, and 2 strong signals. The detected signals were compared with the drug labels used in three countries.

None of the 11 neuroprotectants are marketed in the United States, while two, citicoline sodium and edaravone, are marketed in Japan. Shiver, urinary retention, mental disorder, fatigue, hypertension, dilatation of the stomach, and spasms of citicoline sodium were unlabeled AEs in both China and Japan. Fidgety episodes and fever associated with citicoline sodium were labeled AEs in China but unlabeled in Japan. Chest distress associated with citicoline sodium was an unlabeled AE in China but labeled AE in Japan. Rigors, chest distress, eructation, and mental disorder associated with edaravone were also unlabeled AEs in both China and Japan.

Asphyxia, hyperpyrexia, and wheezing associated with monosialotetrahexosylganglioside sodium; hyperpyrexia associated with cerebroprotein hydrolysate; asphyxia and skin flushing associated with troxerutin and cerebroprotein; crying associated with the mouse nerve growth factor; rigors and hyperpyrexia associated with deproteinized calf blood serum; swelling of the head associated with *Ginkgo biloba* leaf extract; swelling of the head, fever, numbness, vertigo, localized numbness, fidgety episodes, and chest distress associated with vinpocetine were unlabeled AEs in China. These drugs were not approved in either the USA or Japan.

The four methods were mainly compared based on the sensitivity, specificity, positive predictive value, negative predictive value, and the Jordan index. These indicators are calculated by comparing the signal values detected using the three methods with those detected using the BCPNN method (Table 5).

**TABLE 5:** Comparison of various signal detection methods (drug-adverse events).

Method	Condition (A ≥ values listed)	Sample size	Sensitivity	Specificity	Positive predictive value	Negative predictive value	Jorden index
ROR	3	286	1.00	0.96	0.83	1.00	0.96
	4	221	1.00	0.98	0.90	1.00	0.98
	5	172	1.00	0.99	0.94	1.00	0.99
	6	148	1.00	0.99	0.97	1.00	0.99
	7	132	1.00	0.99	0.96	1.00	0.99
	11	92	1.00	1.00	1.00	1.00	1.00
PRR	3	286	1.00	0.96	0.83	1.00	0.96
	4	221	1.00	0.98	0.90	1.00	0.98
	5	172	1.00	0.99	0.94	1.00	0.99
	6	148	1.00	0.99	0.97	1.00	0.99
	7	132	1.00	0.99	0.96	1.00	0.99
	11	92	1.00	1.00	1.00	1.00	1.00
MHRA	3	286	0.73	1.00	0.97	0.95	0.72
	4	221	0.89	0.99	0.97	0.98	0.88
	5	172	0.88	0.99	0.97	0.97	0.87
	6	148	0.86	1.00	1.00	0.97	0.86
	7	132	0.83	1.00	1.00	0.96	0.83
	11	92	0.81	1.00	1.00	0.95	0.81

## 4 Discussion

In this study, we detected 84 signals associated with neuroprotectants by analyzing the spontaneous AE reporting system database reported to the Jinan AE reporting system from 2000 to 2022. Fifty-four signal-related AEs are already listed in the drug instructions.

Citicoline sodium, which had the most unlabeled AEs, is one of the most widely used neuroprotectants. There are nine adverse reaction signals that are not listed on drug labels used in China, and two are not listed on those used in Japan. Chest distress has been included on drug labels in Japan. The pathogenesis of mental disorder and fidgety episodes is not fully clear (Ang et al., 2016). Chest distress is not listed on drug labels used in China but is listed on those used in Japan. Detection of this signal can improve drug labeling.

Hyperpyrexia associated with cerebroprotein hydrolysate is one of the known systemic effects. The mechanism of this AE is, at the time of writing, unknown. Clinical treatment for patients with a hypersensitive physique must be observed at any time; once adverse reactions occur, the drug must be discontinued timeously, and symptomatic treatment administered to prevent and reduce harm caused by adverse reactions to the human body (Tian et al., 2014).

Asphyxia is one of the symptoms of a suspected allergic reaction caused by troxerutin and cerebroprotein. This drug is a sterilized aqueous solution of extracts. Due to the production process and other factors, if the extract is not pure, ADRs are likely to occur; this drug also contains protein, which can act as an antigen or hapten substance that enters the body, stimulating the production of antibodies or sensitized lymphocytes, resulting in an immune response (Ying et al., 2011; Zhang and Huang, 2017).

Rigors and hyperpyrexia are the symptoms of a suspected allergic reaction caused by deproteinized calf blood serum. This drug, as a hapten, can be combined with biological macromolecules, such as proteins, in the body to form a complete antigen, stimulating the body to produce antibodies and causing allergic reactions, especially for individual patients with particular physical conditions (Sun and Li, 2015). Biotherapeutic agents were predominantly associated with hypersensitivity-related ADRs, necessitating enhanced clinical monitoring and emergency preparedness during administration.

Numbness and localized numbness associated with use of vinpocetine may be related to its high fat-solubility, easy passage through the blood–brain barrier, and increased effect of cyclophosphosphate guanosine (c-GMP), a messenger of vascular smooth muscle relaxation (Zhang et al., 2016).

Abnormal liver function associated with the use of edaravone is a moderate-intensity signal. The same research result was also found in the study by Qi Shang et al. They found that the impaired hepatic function may be associated with increased plasma concentrations of the prototype edaravone (Shang et al., 2025). Monosialotetrahexosylganglioside sodium demonstrated the strongest signal intensity for chest distress and fever and low signal intensity for dyspnea, potentially attributable to allergic reaction. The raw materials of this drug were derived from animal organs. The manufacturing process involves multiple steps, such as lipid extraction, hydrolysis, and dialysis, using large amounts of organic solvents and polymer substances. The complex preparation process is a major reason why it is prone to causing adverse reactions, including allergies (Zhang and Chen, 2024). Edaravone was associated with abnormal liver function in our dataset. Although some preclinical studies suggest hepatoprotective or anti-inflammatory effects (Zhao et al., 2022), human data on hepatic safety are limited and conflicting. Clinicians should consider monitoring liver function in patients with underlying hepatic conditions.

The mechanism underpinning other new adverse reactions has not yet been reported. Further clinical studies to confirm the mechanism are necessary to ensure drug safety.

Drugs such as monosialotetrahexosylganglioside sodium, cerebroprotein hydrolysate, troxerutin and cerebroprotein, mouse nerve growth factor, deproteinized calf blood serum, extract of *Ginkgo biloba* leaves, and vinpocetine have not yet been approved for marketing abroad but are widely applied in large quantities in China for neuroprotection, which poses a safety risk. The risk signals identified in this study that are not listed on the labels are valuable for further research on the regulation of post-marketing drug safety.

PRR and ROR methods showed better sensitivity, and MHRA methods had better specificity than BCPNN. The signals which were detected using PRR and ROR methods but not using MHRA and BCPNN methods might have a higher probability of being false positive ; more emphasis should be placed on this type of signal analysis and confirmation.

The present study sheds light on critical unmet needs in neuroprotectant safety profiling, particularly for citicoline sodium. Eight new citicoline, seven new vinpocetine, four new monosialotetrahexosylganglioside sodium, and four new edaravone-associated AEs not listed on the Chinese label were identified. These results could inform drug safety updates and label revisions, particularly in China, where citicoline is commonly used. Bridging labeling disparities requires harmonizing international pharmaco-vigilance standards and adopting advanced analytics (e.g., federated learning across Asian AE databases). Clinically, the present findings advocate for risk-stratified dosing protocols and enhanced production quality controls to mitigate systemic and neuropsychiatric risks.

There are some limitations in this study. First, the Jinan AE reporting system is a self-reporting database, and there is the possibility of missing reports. The quantitative signal detection method adopted in the present study is based on the quantitative correlation of the Jinan AE reporting system data. The results only indicate that there is a certain correlation between drugs and AEs, but not necessarily a causal relationship between drugs and AEs. In addition, this study only focused on a single drug–AE combination, without considering the factors of drug combination. Individual adverse reactions may result from the interaction of multiple drugs, which needs to be further determined through manual evaluation and analysis. The predominance of China-specific agents (e.g., cerebroprotein hydrolysate) limits direct comparability with international safety profiles. For 30% of newly detected signals (e.g., citicoline-induced mental disorders), proposed mechanisms remain hypothetical without experimental validation. The antigen–hapten theory for protein-based agents requires *in vitro*/*in vivo* confirmation.

Our study has several strengths: the safety evidence gap for China-specific neuroprotectants: this study systematically identified unlabeled safety signals (e.g., nine citicoline-related signals absent from Chinese labels) through spontaneous reporting system analysis (2000–2022) for neuroprotective agents (e.g., monosialotetrahexosylganglioside sodium, cerebroprotein hydrolysate, troxerutin, and cerebroprotein) that are widely used in China but not approved internationally, thereby filling critical safety evidence gaps in the global literature. Label disparity mechanistic insights: the present results revealed clinically significant drug label discrepancies between China and Japan (e.g., citicoline-induced “chest distress” being documented on Japanese but not on Chinese labels) and provided pathophysiological rationale (e.g., the antigen–hapten theory for hyperpyrexia induced by cerebroprotein hydrolysate) to support updates to labels.

## 5 Conclusion

PRR, ROR, MHRA, and BCPNN signal detection methods were adopted to discover some of the high-intensity and new AE signals caused by 11 neuroprotective agents, increasing the types of adverse reactions from neuroprotective agents and providing reference for further evaluation of the clinical safe and rational use of drugs and drug-safety risks. We detected signals of neuroprotective agents and compared this information across the labels used in two countries; we found that 30 AEs were not included on the drug labels used in China, 11 AEs were not included on those used in Japan, and 2 AEs were included on the drug labels used in China but not in Japan. The AE signals detected through the ratio imbalance measure were derived from a statistical correlation of the frequency of AE reports. Further investigation and evaluation are required to determine the potential biological relationship between drugs and AEs. This nationwide pharmacovigilance study represents one of the most comprehensive safety evaluations of neuroprotective agents conducted in China so far. By integrating multi-year, population-scale adverse event data, our analysis not only reveals previously unrecognized safety signals but also establishes a robust evidence base with high external validity. The longitudinal design and national coverage of this study enhance its translational value and drug safety policymaking.

## Data Availability

The original contributions presented in the study are included in the article/Supplementary Material; further inquiries can be directed to the corresponding authors.
