# Supplementary material for: Mining and evaluation of security alert signals of neuroprotective agents based on the Jinan adverse event reporting system database: a retrospective study
Source: Front Pharmacol. 2025 Aug 5;16:1529923. doi: 10.3389/fphar.2025.1529923 (PMC12361204; doi:10.3389/fphar.2025.1529923)
Supplement: Supplementary file 1 [file Table1.docx]

**Supplemental Table 1.** Detected signals for neuroprotective agents by data mining and existence of the information on the drug labels

|  |  |  |  |  |  |  | **Drug label** | | |
| --- | --- | --- | --- | --- | --- | --- | --- | --- | --- |
| **Drugs** | **Adverse events** | **No. of AE reports** | **PRR** | **ROR** | **MHRA** | **BCPNN** | **China** | **USA** | **Japan** |
| Oxiracetam |  |  |  |  |  |  |  |  |  |
|  | Rigors | 27 | 1.97 | 2.03 | 12.23* | 0.92^a^ | Y | NA | NA |
|  | Infusion-related reactions | 3 | 8.59 | 8.64 | 12.95* | 1.56^a^ | Y | NA | NA |
|  | Insomnia | 12 | 5.97 | 6.11 | 44.34 | 2.1^a^ | Y | NA | NA |
|  | Hepatic function abnormal | 11 | 3.04 | 3.09 | 13.07 | 1.37^a^ | Y | NA | NA |
|  | Euphoria | 6 | 14.84 | 15.03 | 61.82 | 2.3^a^ | Y | NA | NA |
|  | Spasm | 6 | 4.81 | 4.86 | 14.35 | 1.63^a^ | Y | NA | NA |
|  | Upset stomach | 3 | 3.82 | 3.84 | 3.71* | 1.16* | Y | NA | NA |
|  | Fever | 18 | 30.46 | 31.71 | 448.63 | 3.53^b^ | Y | NA | NA |
|  | Chest distress | 17 | 7.55 | 7.82 | 88.91 | 2.45^b^ | Y | NA | NA |
|  | Mental disorder | 13 | 10.47 | 10.75 | 99.57 | 2.62^b^ | Y | NA | NA |
| Citicoline sodium |  |  |  |  |  |  |  |  |  |
|  | Shiver | 3 | 92.13 | 92.58 | 140.03* | 1.93^a^ | N | NA | N |
|  | Urinary retention | 3 | 7.61 | 7.64 | 10.93* | 1.51^a^ | N | NA | N |
|  | Mental disorder | 12 | 7.01 | 7.13 | 54.74 | 2.24^a^ | N | NA | N |
|  | Upset stomach | 9 | 8.32 | 8.43 | 49.45 | 2.24^a^ | Y | NA | Y |
|  | Fidgety | 7 | 3.18 | 3.2 | 8.31 | 1.31^a^ | Y | NA | N |
|  | Fatigue | 6 | 38.56 | 38.94 | 161.04 | 2.57^a^ | N | NA | N |
|  | Hypertension | 4 | 5.64 | 5.67 | 10.76 | 1.53^a^ | N | NA | N |
|  | Headache | 4 | 122.83 | 123.64 | 255.36 | 2.25^a^ | Y | NA | Y |
|  | Suppression of appetite | 3 | 3.64 | 3.65 | 3.35* | 1.12* | Y | NA | Y |
|  | Dilatation of the stomach | 3 | 5.38 | 5.41 | 6.64* | 1.35* | N | NA | N |
|  | Spasm | 5 | 2.91 | 2.93 | 4.46 | 1.13* | N | NA | N |
|  | Insomnia | 37 | 13.37 | 14.17 | 393.94 | 3.28^b^ | Y | NA | Y |
|  | Fever | 21 | 25.8 | 26.68 | 435.94 | 3.54^b^ | Y | NA | N |
|  | Chest distress | 20 | 6.45 | 6.63 | 85.17 | 2.33^b^ | N | NA | Y |
|  | Gastrointestinal reactions | 19 | 6.72 | 6.9 | 85.11 | 2.36^b^ | Y | NA | Y |
|  | Euphoria | 10 | 17.95 | 18.23 | 134.85 | 2.79^b^ | Y | NA | Y |
| Monosialotetrahexosylganglioside sodium |  |  |  |  |  |  |  |  |  |
|  | Chest distress | 87 | 19.53 | 21.57 | 1379.26 | 3.9^c^ | Y | NA | NA |
|  | Fever | 30 | 25.65 | 26.52 | 606.25 | 3.74^c^ | Y | NA | NA |
|  | Flushing | 34 | 1.79 | 1.83 | 11.29* | 0.8^a^ | Y | NA | NA |
|  | Fatigue | 3 | 13.42 | 13.46 | 21.63* | 1.69^a^ | Y | NA | NA |
|  | Rigors | 65 | 2.39 | 2.51 | 52.35 | 1.22^a^ | Y | NA | NA |
|  | Asphyxia | 29 | 2.24 | 2.28 | 18.73 | 1.09^a^ | N | NA | NA |
|  | Hyperpyrexia | 23 | 2.42 | 2.46 | 17.74 | 1.18^a^ | N | NA | NA |
|  | Numbness | 9 | 2.64 | 2.66 | 7.52 | 1.17^a^ | Y | NA | NA |
|  | Shortness of breath | 8 | 2.81 | 2.83 | 7.53 | 1.21^a^ | Y | NA | NA |
|  | Infusion-related reactions | 4 | 5.79 | 5.81 | 11.06 | 1.54^a^ | Y | NA | NA |
|  | Wheezing | 4 | 4.93 | 4.95 | 8.68 | 1.45^a^ | N | NA | NA |
|  | Dyspnoea | 30 | 1.45 | 1.46 | 3.77* | 0.51* | Y | NA | NA |
|  | Shiver | 6 | 128.24 | 129.12 | 381.02 | 2.69^b^ | N | NA | NA |
| Cerebroprotein Hydrolysate |  |  |  |  |  |  |  |  |  |
|  | Rigors | 16 | 4.37 | 4.9 | 39.37 | 1.85^a^ | Y | NA | NA |
|  | Palpitation | 10 | 2.58 | 2.73 | 8.42 | 1.16^a^ | Y | NA | NA |
|  | Hyperpyrexia | 7 | 5.46 | 5.74 | 21.4 | 1.8^a^ | N | NA | NA |
|  | Fever | 10 | 63.4 | 69.17 | 530.45 | 3.24^b^ | Y | NA | NA |
| Troxerutin- cerebroprotein complex |  |  |  |  |  |  |  |  |  |
|  | Asphyxia | 12 | 1.93 | 1.96 | 4.54* | 0.84^a^ | N | NA | NA |
|  | Fatigue | 3 | 27.96 | 28.15 | 49.84* | 1.84^a^ | Y | NA | NA |
|  | Low back pain | 3 | 6.75 | 6.8 | 9.36* | 1.46^a^ | Y | NA | NA |
|  | Skin flushing | 6 | 3.75 | 3.79 | 9.43 | 1.42^a^ | N | NA | NA |
|  | Tic | 4 | 3.37 | 3.4 | 4.49 | 1.19* | Y | NA | NA |
|  | Arrhythmia | 4 | 3.96 | 3.99 | 6.08 | 1.31* | Y | NA | NA |
|  | Chest distress | 26 | 12.16 | 12.89 | 248.97 | 3.07^b^ | Y | NA | NA |
|  | Fever | 9 | 16.03 | 16.36 | 108.04 | 2.66^b^ | Y | NA | NA |
| Mouse nerve growth factor |  |  |  |  |  |  |  |  |  |
|  | Cry | 4 | 33.89 | 36.01 | 95.8 | 2.16^a^ | N | NA | NA |
|  | Pain | 3 | 3.49 | 3.61 | 3.17* | 1.09* | Y | NA | NA |
|  | Injection site pain | 12 | 12.55 | 15.12 | 117.43 | 2.72^b^ | Y | NA | NA |
| Deproteinised calf blood serum |  |  |  |  |  |  |  |  |  |
|  | Fever | 3 | 27.37 | 28.37 | 51.5* | 1.85^a^ | Y | NA | NA |
|  | Rigors | 8 | 3.14 | 3.38 | 9.94 | 1.33^a^ | N | NA | NA |
|  | Flushing | 6 | 3.38 | 3.56 | 7.96 | 1.32^a^ | Y | NA | NA |
|  | Hyperpyrexia | 3 | 3.37 | 3.46 | 2.93* | 1.07* | N | NA | NA |
| Edaravone |  |  |  |  |  |  |  |  |  |
|  | Rigors | 12 | 1.97 | 2.04 | 4.97* | 0.87^a^ | N | NA | N |
|  | Chest distress | 9 | 9.02 | 9.4 | 56.09 | 2.31^a^ | N | NA | N |
|  | Injection site pruritus | 6 | 7.67 | 7.88 | 28.29 | 1.96^a^ | Y | NA | Y |
|  | Fever | 5 | 19.08 | 19.56 | 67.13 | 2.24^a^ | Y | NA | Y |
|  | Eructation | 4 | 20.09 | 20.49 | 53.5 | 2.05^a^ | N | NA | N |
|  | Mental disorder | 3 | 5.45 | 5.52 | 6.87* | 1.36* | N | NA | N |
|  | Increased blood pressure | 3 | 3.95 | 4.0 | 3.99* | 1.18* | Y | NA | Y |
|  | Hepatic function abnormal | 16 | 9.96 | 10.76 | 119.82 | 2.69^b^ | Y | NA | Y |
| Extract of ginkgo biloba leaves |  |  |  |  |  |  |  |  |  |
|  | Superficial phlebitis | 3 | 23.37 | 23.83 | 42.95* | 1.82^a^ | Y | NA | NA |
|  | Increased dizziness | 3 | 44.89 | 45.8 | 85.24* | 1.9^a^ | Y | NA | NA |
|  | Upset stomach | 3 | 11.41 | 11.63 | 18.87* | 1.66^a^ | Y | NA | NA |
|  | Rigors | 11 | 2.39 | 2.51 | 7.82 | 1.09^a^ | Y | NA | NA |
|  | Phlebitis | 7 | 3.47 | 3.59 | 10.05 | 1.4^a^ | Y | NA | NA |
|  | Swelling of the head | 4 | 2.69 | 2.74 | 2.76* | 1.0* | N | NA | NA |
|  | Chest distress | 12 | 15.92 | 17.24 | 151.91 | 2.88^b^ | Y | NA | NA |
|  | Fever | 8 | 40.44 | 42.69 | 260.59 | 2.9^b^ | Y | NA | NA |
| Vinpocetine |  |  |  |  |  |  |  |  |  |
|  | Flutter of the heart | 11 | 2.27 | 2.32 | 6.67 | 1.03^a^ | Y | NA | NA |
|  | Swelling of the head | 8 | 2.71 | 2.76 | 7.06 | 1.18^a^ | N | NA | NA |
|  | Fever | 6 | 15.27 | 15.56 | 64.7 | 2.32^a^ | N | NA | NA |
|  | Fatigue | 5 | 66.57 | 67.7 | 233.5 | 2.47^a^ | Y | NA | NA |
|  | Numbness | 5 | 4.36 | 4.42 | 9.78 | 1.48^a^ | N | NA | NA |
|  | Vertigo | 5 | 5.75 | 5.83 | 15.04 | 1.67^a^ | N | NA | NA |
|  | Fatigue | 4 | 11.62 | 11.77 | 28.39 | 1.89^a^ | Y | NA | NA |
|  | Localized numbness | 5 | 2.4 | 2.43 | 2.82* | 0.96* | N | NA | NA |
|  | Fidgety | 4 | 3.76 | 3.8 | 5.56 | 1.27* | N | NA | NA |
|  | Chest distress | 14 | 9.35 | 9.77 | 95.18 | 2.57^b^ | N | NA | NA |

AEs, adverse events; PRR, proportional reporting ratios; ROR, reporting odds ratios; BCPNN, Bayesian Confidence Propagation Neural Network; MHRA, Medicines and Healthcare products Regulatory Agency; N, AE is not included in drug label; Y, AE is included in drug label; NA, not approved; *, there is no signal for this method; a, weak signal for BCPNN method; b, medium signal for BCPNN method; c, strong signal for BCPNN method.
